# The BET inhibitor apabetalone decreases neuroendothelial proinflammatory activation *in vitro* and in a mouse model of systemic inflammation

**DOI:** 10.1515/tnsci-2022-0332

**Published:** 2023-12-31

**Authors:** Sylwia Wasiak, Li Fu, Emily Daze, Dean Gilham, Brooke D. Rakai, Stephanie C. Stotz, Laura M. Tsujikawa, Chris D. Sarsons, Deborah Studer, Kristina D. Rinker, Ravi Jahagirdar, Norman C. W. Wong, Michael Sweeney, Jan O. Johansson, Ewelina Kulikowski

**Affiliations:** Resverlogix Corp., Suite 300, 4820 Richard Road SW, Calgary, AB, T3e 6L1, Canada; Department of Biomedical Engineering, Department of Physiology and Pharmacology, Libin Cardiovascular Institute, University of Calgary, 2500 University Dr. NW, Calgary, AB, T2N 1N4, Canada; Resverlogix Corp., 535 Mission Street, 14th Floor, San Francisco, CA, 94105, USA

**Keywords:** blood–brain barrier, epigenetics, inflammation, bromodomain, microvascular disease

## Abstract

Brain vascular inflammation is characterized by endothelial activation and immune cell recruitment to the blood vessel wall, potentially causing a breach in the blood – brain barrier, brain parenchyma inflammation, and a decline of cognitive function. The clinical-stage small molecule, apabetalone, reduces circulating vascular endothelial inflammation markers and improves cognitive scores in elderly patients by targeting epigenetic regulators of gene transcription, bromodomain and extraterminal proteins. However, the effect of apabetalone on cytokine-activated brain vascular endothelial cells (BMVECs) is unknown. Here, we show that apabetalone treatment of BMVECs reduces hallmarks of *in vitro* endothelial activation, including monocyte chemoattractant protein-1 (MCP-1) and RANTES chemokine secretion, cell surface expression of endothelial cell adhesion molecule VCAM-1, as well as endothelial capture of THP-1 monocytes in static and shear stress conditions. Apabetalone pretreatment of THP-1 downregulates cell surface expression of chemokine receptors CCR1, CCR2, and CCR5, and of the VCAM-1 cognate receptor, integrin α4. Consequently, apabetalone reduces THP-1 chemoattraction towards soluble CCR ligands MCP-1 and RANTES, and THP-1 adhesion to activated BMVECs. In a mouse model of brain inflammation, apabetalone counters lipopolysaccharide-induced transcription of endothelial and myeloid cell markers, consistent with decreased neuroendothelial inflammation. In conclusion, apabetalone decreases proinflammatory activation of brain endothelial cells and monocytes *in vitro* and in the mouse brain during systemic inflammation.

## 1 Introduction

In healthy conditions, brain microvascular endothelial cells (BMVECs) form a tight monolayer that insulates the brain parenchyma from blood [1]. However, during systemic or brain inflammatory disease, blood- and brain-derived cytokines stimulate BMVECs to secrete vascular endothelial growth factors and proinflammatory molecules that increase the monolayer permeability to molecules and proteins [1,2]. Proinflammatory factors also upregulate BMVEC surface expression of cell adhesion molecules (CAMs), selectins and chemokines that promote monocyte recruitment to the neuro-endothelium [2,3] as well as their differentiation into proinflammatory macrophages upon brain entry [4]. BMVEC-derived signals also activate brain-resident cells, including pericytes, astrocytes, and microglia, impacting blood–brain barrier (BBB) permeability [4]. A complex crosstalk between infiltrating macrophages and brain-resident cells leads to the release of cytokines, including interleukin (IL) 1β, IL-6, IL-8, tumor necrosis factor (TNF) α, monocyte chemoattractant protein-1 (MCP-1) and macrophage inflammatory protein (MIP)-1α, reactive oxygen species, and proteolytic enzymes, all of which contribute to neuroinflammation, and ultimately, neurodegeneration and cognitive decline [5].

Endothelial response to cytokines relies on the epigenetic reader BRD4, a bromodomain and extraterminal (BET) protein that initiates proinflammatory gene transcription by binding to acetylated lysine residues on histones and transcription factors [5]. Through its bromodomains (BD) 1 and 2, BRD4 binds to acetylated nuclear factor κ-light-chain-enhancer of activated B cells (NF-κB) [6], leading to RNA polymerase II activation and transcription of canonical proinflammatory genes that characterize endothelial activation, including chemokines, CAMs and selectins [7]. Small-molecule BET inhibitors (BETi) that target BET BDs, including pan-BD inhibitor JQ1 [8] and BD2-selective apabetalone [9], have been shown to reduce proinflammatory gene expression in cytokine-activated human umbilical vein endothelial cells (HUVECs) [7,10,11], human aortic endothelial cells (HAECs) [7,10], and pulmonary endothelial cells [12]. Functionally, BETi treatment countered *in vitro* monocyte adhesion to TNFα-activated HAECs [10], HUVECs [7], and to the mouse cremaster muscle postcapillary venules [7]. Further, in aged mice, BET degradation with the proteolysis targeting chimera (PROTAC) dBET1 mitigated BBB damage induced by focal cerebral ischemia, prevented infiltration of neutrophils, and reduced brain levels of proinflammatory mediators [13]. BET proteins have also been shown to regulate angiogenesis [6,14] and endothelial–mesenchymal transition [15,16]. These data indicate that BETs play a widespread role in endothelial functions and that inhibiting their activity may be beneficial for the treatment of conditions characterized by vascular inflammation, including neuroinflammatory diseases.

Apabetalone (RVX-208) is a clinical-stage BETi that has shown promise in attenuating cardiovascular disease (CVD) in patients with type-2 diabetes mellitus (T2DM) [17,18]. Interestingly, apabetalone treatment also improved the cognition score of CVD patients with T2DM and cognitive impairment [19]. Given apabetalone’s *in vitro* and *in vivo* activity in vascular pathology, we hypothesized that apabetalone might act by reducing endothelial inflammation in the brain. Here, we investigated the impact of apabetalone on proinflammatory activation of BMVECs and their interactions with monocytes using *in vitro* models of vascular inflammation and a mouse model of brain inflammation. Our data indicate that targeting BET proteins with epigenetic therapeutics reduces neuroendothelial proinflammatory activation.

## 2 Materials and methods

### 2.1 Chemical synthesis

Apabetalone and JQ1 were synthesized by NAEJA Pharmaceuticals (Edmonton, Canada) or IRIX Pharmaceuticals (Florence, SC) [20]. The PROTAC MZ1 was obtained from Tocris Bioscience.

### 2.2 Tissue culture

Primary human BMVECs were obtained from Cell Systems^®^ and plated on collagen-coated flasks in a complete classic medium with 10% serum and CultureBoost™ as recommended (passage 3). After two passages, cells were cryopreserved in Cell Systems’ cell freezing medium and used for experiments between passages 5 and 7. hCMEC/D3 cells (Millipore Sigma) were grown on collagen-coated flasks in EndoGRO-MV Complete Culture Media Kit with 1 ng/mL bFGF (Millipore Sigma). THP-1 cells (ATCC^®^) were cultured in ATCC^®^-modified RPMI-1640 medium with 10% heat-inactivated FBS (Canada origin), 1× Gibco™ penicillin–streptomycin (ThermoFisher Scientific), 5 µg/mL Plasmocin™ (InvivoGen) and 0.05 mM β-mercaptoethanol (Millipore Sigma) and used between passages 10 and 35. All cells were incubated at 37°C in a humidified atmosphere enriched with 5% CO_2_.

### 2.3 Quantification of mRNA expression

hCMEC/D3 and BMVECs were treated with 10 or 100 ng/mL TNFα + IFNγ (StemCell Technologies) ± BETi or dimethyl sulfoxide (DMSO) for 4–24 h. In 4 h time-point experiments, cells were pre-incubated with BETi for 1 h prior to stimulation, and in 24 h time-point experiments, all treatments were applied simultaneously. mRNA isolation and analysis were done as previously described [21].

### 2.4 Flow cytometry

Primary BMVECs were stimulated for 4–18 h with 10 ng/mL TNFα + IFNγ ± BETi or DMSO, followed by staining with FITC anti-VCAM1 and APC anti-E-selectin antibodies (BD™ Bioscience). THP-1 were treated with DMSO or BETi for 48 h followed by staining with anti-CCR1 Alexa Fluor^®^ 647, anti-CCR2 PE, anti-CCR5 FITC, anti-CXCR2 FITC, anti-ITGA4 BV421 or anti-IGTAM APC antibodies or isotype controls (BD™ Bioscience). Fluorescence was quantified using BD FACSCelesta™ Flow Cytometer. Mean fluorescence intensity (MFI) and % positive cell numbers were calculated with FlowJo™. The concentrations of MCP-1 and IL-6 in the tissue culture supernatant were measured using a BD™ Cytometric Bead Array Flex Set.

### 2.5 Endothelial monolayer permeability and protein secretion

hCMEC/D3 cells were plated in Vascular Permeability Assay kit plates (24-well) (Millipore Sigma) at 50,000 or 100,000 cells/filter (day 1). Media was changed on days 3 and 6. On day 7, cells received 0.025% DMSO, 100 ng/mL TNFα + IFNγ and 25 μM apabetalone. Monolayer permeability was measured as per the manufacturer’s instructions. Tissue culture supernatants were analyzed using a Milliplex^®^ Human Cytokine/Chemokine Array 42-Plex with IL-18 (HD42) (Eve Technologies, Calgary, AB).

### 2.6 Chemoattraction assay

Corning^®^ Transwell^®^ polycarbonate membrane cell culture inserts (6-well plates, Millipore Sigma) were coated in EmbryoMax^®^ 0.1% gelatin (Millipore Sigma) and air-dried. The bottom chambers were filled with M199 + 0.1% human serum albumin (HSA) (Millipore Sigma) (2.5 mL/well) ± 40 ng/mL MCP-1 or 100 ng/mL RANTES (R&D Systems). THP-1 cells pre-treated with 0.025% DMSO or 25 µM apabetalone for 48 h were placed in top chambers at 3 million cells/2.5 mL of media and allowed to transmigrate overnight. Media in both chambers contained either 0.025% DMSO or 25 µM apabetalone. Cells were retrieved from the bottom chamber by centrifugation at 120 *g* for 5 min in a microfuge, stained with 0.4% Trypan Blue Solution (ThermoFisher Scientific), and counted with an Invitrogen™ Countess™ Automated Cell Counter.

### 2.7 Static cell adhesion assay

hCMEC/D3 cells were seeded in 100 µL/well of media at 30,000 cells/well in black/clear 96-well optical-bottom collagen-coated plates (ThermoFisher Scientific). At 48 h, cells were washed in 1× M199 (ThermoFisher Scientific) + 0.1% HSA, pre-incubated in media ± 5–25 µM apabetalone for 30 min prior to addition of 10 ng/mL TNFα + IFNγ for 4 h. THP-1 cells were treated with 0.025% DMSO or 25 µM apabetalone for 48 h, washed in DPBS (with Ca^2+^ and Mg^2+^; ThermoFisher Scientific), stained with 5 µM Vybrant™ CFDA SE Cell Tracer Kit (ThermoFisher Scientific) and incubated with hCMEC/D3 monolayers at 100,000 cells per 200 µL of M199 + 0.1% HSA for 30 min at 37°C. Plates were washed four times with 200 μL media, twice with DPBS, fixed in 4% paraformaldehyde (pH 7.8) for 5 min, and then washed twice with DPBS. Florescence from adhering THP-1 cells was quantified with Synergy 4 (excitation: 485 nm; emission: 528 nm). Micrographs were obtained using a Leica DM IL microscope and the Leica Suite Software.

### 2.8 Parallel flow adhesion assay

BMVECs were seeded onto collagen-coated standard glass microscope slides at a density of 0.22 million cells/slide, pretreated with BETi (or DMSO) for 1 h, followed by BETi (or DMSO) + 10 ng/mL cytokines for 4 h. The assay was performed as described by Tsujikawa et al. [10].

### 2.9 Mouse studies

Prior to LPS administration, 8-week-old male C57BL/6 mice received vehicle or apabetalone (150 mg/kg b.i.d., formulation EA006) by gavage for 6 days. On day 7, mice received apabetalone 4 h prior to an intraperitoneal injection of *Escherichia coli* 0111:B4 LPS (10 µg per mouse) (Millipore Sigma), and again at the time of LPS injection. Animals were sacrificed on day 8, 24 h after LPS injection. Brains were harvested, rinsed in PBS, and snap-frozen. RNA extraction was performed as described by Wasiak et al. [21]. Gene expression of 29 genes was examined, including *Itgal*, *Ccr5*, *Ccr2*, *Itga4*, *Cxcr2*, *Cd68*, *Ccl5*, *Sele*, *Icam*, *Selp*, *IL1b*, *Cxcr3*, *Vcam1*, *Tnf*, *IL1rn*, *Aif1*, *Cx3cr1*, *Itgam*, *Csf1*, *Cd14*, *Cx3cl1*, *Ccl12*, *Cxcl2*, *Cd69*, *Marco*, *Ccl2*, *Il17*, *Cxcr4* and *Cxcl10* as described by Wasiak et al. [21]. In the pharmacokinetics study, three non-fasted animals received a single oral dose of 150 mg/kg apabetalone, and plasma and perfused brain were collected at 3 h. The snap-frozen samples were analyzed at Climax Laboratories (San Jose, CA, USA).

### 2.10 Statistical analysis

Statistical significance was calculated with GraphPad Prism software version 10. One-way ANOVA followed by Tukey’s or Dunnett’s multiple comparison test or two-way ANOVA followed by Tukey’s multiple comparison test for within-group comparisons or Bonferroni’s test for between-group comparisons, Student’s *t*-test for comparisons between two groups, with added Holm–Sidak test if multiple comparisons were performed. Results were presented as mean or median values of at least 3 repeats ± standard deviation (SD) or standard error of the mean. *p*-value ≤ 0.05 was considered statistically significant.


**Ethical approval:** The research related to animals’ use complied with all the relevant national regulations and institutional policies for the care and use of animals. Animal studies were performed at Aravasc Inc. (Sunnyvale, CA) following NIH guidelines and NASA Animal Care and Use Committee (IACUC) policy with approved protocol ARA-16-001-Y1.

## 3 Results

### 3.1 BETi reduce transcription of cytokines in human BMVECs

To study the effects of BETi, we used the hCMEC/D3 cell line that closely recapitulates the characteristics of human BMVECs [22]. We stimulated cells *in vitro* with 10 ng/mL IFNγ and TNFα to induce changes in cytokine gene transcription [23,24,25]. As previously published [22], hCMEC/D3 cells responded within 4 h to TNFα and IFNγ (TNFα + IFNγ) stimulation, with increased gene transcription of multiple cytokines (Table 1). At the highest dose used (25 and 0.33 μM, respectively), the BD2-selective BETi apabetalone [9] and the pan-BD JQ1 [8] inhibited the expression of cytokine genes between 72 and 99% (Table 1). Half inhibitory concentrations (IC_50_) varied between 1.2 and 13 μM for apabetalone and 0.02 and 0.12 μM for JQ1. Overall, these data are consistent with the inhibition of nuclear factor-κB (NF-κB) transcriptional activity downstream of TNFα by apabetalone that was previously demonstrated in HUVECs [11] and HAECs [10].

**Table 1:** Cytokine-induced gene expression in hCMEC/D3 cells is reduced by BETi treatment

Target name		TNFα + IFNγ	TNFα + IFNγ + apabetalone		TNFα + IFNγ + JQ1	
Protein	Gene	Fold induction*	IC_50_† (µM)	Maximum inhibition‡ (%)	IC_50_† (µM)	Maximum inhibition‡ (%)
Fractalkine	*CX3CL1*	1,863	1.20	98	0.02	99
GM-CSF	*CSF2*	11	2.20	97	0.04	98
MCP-1	*CCL2*	107	4.47	94	0.06	96
IP-10	*CXCL10*	17,448	4.70	86	0.06	90
RANTES	*CCL5*	32	5.10	77	0.07	76
MCP-3	*CCL7*	115	5.20	92	0.07	94
IL-8	*CXCL8*	9	8.30	74	0.01	81
IL-1β	*IL1B*	15	9.00	87	0.09	93
IL-6	*IL6*	11	13.0	72	0.12	83

*mRNA fold-induction in response to 4 h cytokine treatment (10 ng/mL) was calculated relative to cytokine-naive cells treated with vehicle (0.05% DMSO).

†BETi dose–response curves were used to calculate half inhibitory concentrations (IC_50_).

‡Maximum inhibition was calculated relative to the induced state for 25 µM apabetalone and 0.33 µM JQ1. Representative data of three biological repeats are shown.

To confirm the on-target activity of BETi in hCMEC/D3 cells, we used the MZ-1 PROTAC [26] to reduce the expression of BET proteins BRD2, BRD3, and BRD4. PROTAC treatment for 24 h reduced BRD4 by ∼70%, while BRD2 and BRD3 were less affected (21 and 13%, respectively) (Figure S1a and b). The impact of cytokines, BETi, or MZ-1 on cell viability was negligible at 24 h post-treatment (Figure S1c). The MZ-1 mediated degradation of BET proteins reduced TNFα + IFNγ stimulated expression of cytokine transcripts, similar to 24 h pretreatment with apabetalone (Table 2), confirming on-target activity. Thus, BET protein inhibition counters cytokine-induced proinflammatory transcription in hCMEC/D3 cells.

**Table 2:** Cytokine-induced gene expression in hCMEC/D3 cells is reduced by BET protein degradation

Target name		TNFα + IFNγ	TNFα + IFNγ + MZ-1		TNFα + IFNγ + Apabetalone	
Protein	Gene	Fold induction*	% Inhibition†	*p*-value‡	% Inhibition†	*p*-value‡
MCP-3	*CCL7*	25	91	<0.0001	94	<0.0001
Fractalkine	*CX3CL1*	124	86	<0.0001	91	<0.0001
MCP-1	*CCL2*	6	48	<0.0001	65	<0.0001
RANTES	*CCL5*	24	43	<0.0001	77	<0.0001
IL-6	*IL6*	13	42	<0.0001	75	<0.0001
IL-8	*CXCL8*	5	38	<0.0001	62	<0.0001
IP-10	*CXCL10*	608	23	<0.0001	44	<0.0001
GM-CSF	*CSF2*	2	3	ns	72	0.0006

*mRNA fold-induction in response to 24 h cytokine treatment (10 ng/mL) was calculated relative to cytokine-naive cells treated with a vehicle for the same amount of time (0.05% DMSO).

†Gene expression inhibition was calculated relative to the induced state in cells co-treated with cytokines and 25 µM apabetalone.

‡Statistical significance was calculated with one-way ANOVA with Dunnett’s correction. ns, non-significant. Representative data of three biological repeats are shown.

### 3.2 BETi treatment reduces polarized cytokine secretion in BMVECs

**Figure 1:**
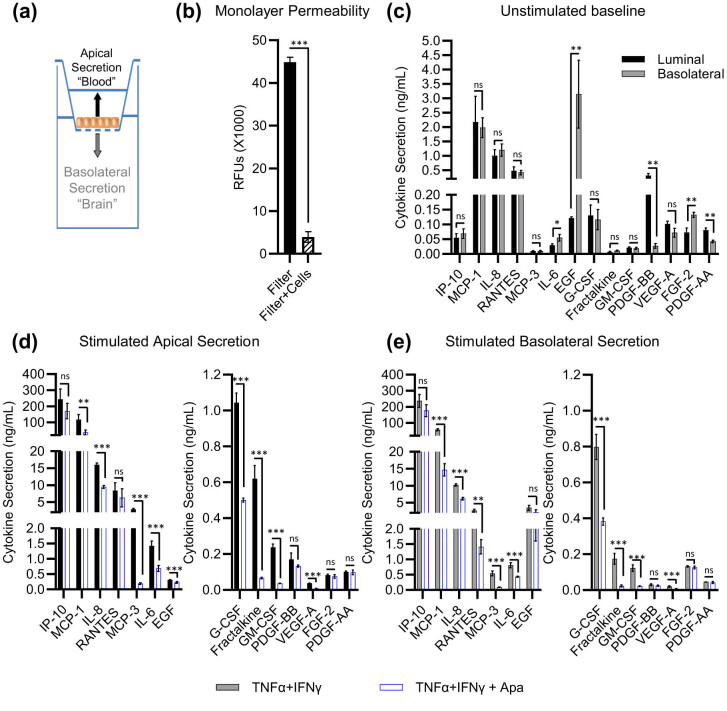
Inducible polarized cytokine secretion by hCMEC/D3 cells is reduced by apabetalone treatment. (a) hCMEC/D3 monolayers grown on hanging cell culture filters secrete cytokines and growth factors into the apical (“blood”) and basolateral (“brain”) compartments. (b) Cell monolayers grown on hanging filters (Filter + Cells) block FITC-dextran diffusion from the apical to the basolateral chamber as compared to filter alone (Filter). Statistical analysis: Student’s *t*-test. (c) At baseline, hCMEC/D3 cell monolayers display a bilateral secretion of cytokines and growth factors as measured by multianalyte immunoprofiling. Protein enrichment in the apical (black bars) or basolateral (grey bars) compartment confirms monolayer impermeability. Statistical analysis: Student’s *t*-test with Holm–Sidak multiple comparison test. (d and e) Stimulation with 100 ng/mL TNFα + IFNγ for 24 h elicits robust secretion of proinflammatory cytokines by hCMEC/D3 cells in both apical (d) and basolateral (e) compartments as measured by multianalyte immunoprofiling (black bars). Co-treatment with 25 μM apabetalone reduces cytokine secretion (grey bars). Statistical analysis: one-way ANOVA with Dunnett’s multiple comparison test. *p ≤ 0.05; **p ≤ 0.01; ***p ≤ 0.001, ns, non-significant. A mean ± standard deviation of *n* = 4 is shown.

Activated BMVECs secrete cytokines and growth factors from both apical and basolateral membranes into blood and brain parenchyma, respectively [27,28]. To study polarized secretion *in vitro*, hCMEC/D3 cells were plated on hanging inserts and allowed to form a monolayer, which enabled quantification of cytokines secreted into the apical (top) or basolateral (bottom) compartment over 24 h (Figure 1a). The cell monolayer was impermeable to high-molecular-weight dextran-fluorescein isothiocyanate (FITC) applied to the apical compartment, demonstrating stable endothelial cell junctions (Figure 1b). Unstimulated endothelial cells secreted cytokines and growth factors to either side of the monolayer, with a few notable exceptions (Figure 1c). Epidermal growth factor was enriched 26-fold in the basolateral compartment, whereas platelet-derived growth factor BB was enriched 11-fold in the apical compartment, indicating polarization of protein secretion across the endothelial monolayer in agreement with a previous report [28]. Apical addition of 100 ng/mL TNFα + IFNγ to the endothelial monolayer elicited robust cytokine gene expression (Table S1), which was 3–25 higher as compared to the treatment with 10 ng/mL TNFα + IFNγ (Table 2). High cytokine concentrations did not alter cell viability after 24 h of treatment (Figure S1c). Consistent with gene expression, protein secretion was also strongly upregulated in both compartments (compare analyte levels in Figure 1c with d for apical secretion, and Figure 1c with e for basolateral secretion). Apabetalone cotreatment substantially reduced cytokine secretion into both apical and basolateral compartments (Figure 1d and e, respectively), indicating that inhibition of BET activity in BMVECs can counter proinflammatory signals produced on both sides of the neuroendothelial monolayer *in vitro*.

### 3.3 BETi-treated BMVECs display lower CAM levels and monocyte adhesion

**Figure 2:**
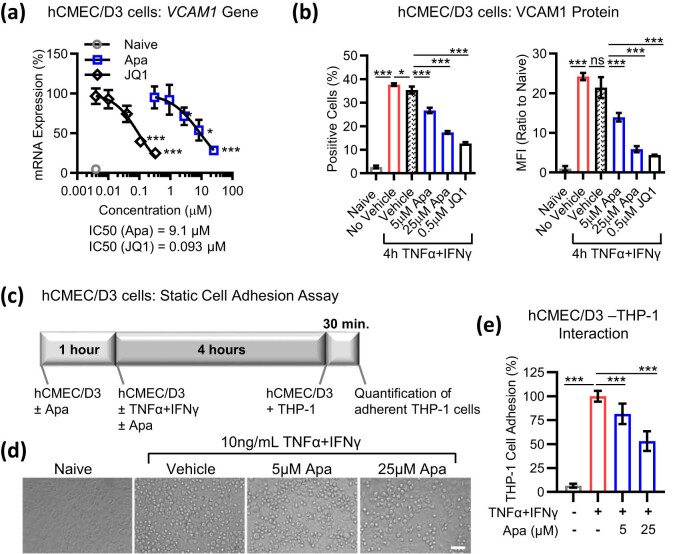
BETi reduce cytokine-induced CAM levels and monocyte adhesion to hCMEC/D3 cells. hCMEC/D3 cells were pre-treated with various concentrations of apabetalone or JQ1 for 1 h prior to incubation with 10 ng/mL TNFα + IFNγ for 4 h, followed by (a) gene expression analysis (real-time PCR) or (b) surface expression analysis (flow cytometry) of VCAM-1. Percentage of cells positive for VCAM-1 and mean fluorescence intensity (MFI) values observed in naive or cytokine-treated conditions are shown. Grey circles or bars represent the non-stimulated (naive) state. DMSO was used as the compound vehicle. Statistical analysis: one-way ANOVA with Dunnett’s multiple comparison test. (c) Experimental protocol for THP-1 cell adhesion to hCMEC/D3 cell monolayers in static (non-flow) conditions. (d) Phase-contrast micrographs show THP-1 adhesion to hCMEC/D3 in the absence (naive) or presence of 10 ng/mL TNFα + IFNγ, vehicle (DMSO), or apabetalone. Scale bar, 100 μm. (e) Quantification of microscopy shows a reduction of cytokine-induced THP-1 cell adhesion to hCMEC/D3 monolayers in the presence of apabetalone (average of six images/condition of three experimental replicates). Statistical analysis: one-way ANOVA with Dunnett’s multiple comparison test. *p ≤ 0.05; ***p ≤ 0.001, ns, non-significant.

Upregulation of surface cell adhesion proteins, including CAMs, selectins, and integrins, is a hallmark of TNFα-mediated activation of endothelial cells [2]. In hCMEC/D3 cells, a 4 h TNFα + IFNγ treatment induced *VCAM1* gene expression (Figure 2a, where the grey circle shows basal expression and curves show cytokine-induced expression). Increasing doses of apabetalone or JQ1 countered this induction with half inhibitory concentrations (IC_50_) of 9 and 0.09 μM, respectively. VCAM-1 protein abundance was also downregulated by BETi treatment, resulting in a reduced number of VCAM-1-positive cells and lower VCAM-1 surface levels (Figure 2b; grey bars show basal expression in naive cells and red bars show cytokine-induced expression).

Firm adhesion of monocytes to vascular endothelial cells is modulated by multiple TNFα target molecules, including VCAM-1 [29,30]. First, hCMEC/D3 cells were pretreated with apabetalone for 1 h prior to 4 h cytokine stimulation (Figure 2c). Fluorescently labeled THP-1 cells were allowed to adhere to activated hCMEC/D3 cells for 30 min, followed by signal quantification (Figure 2c). As expected, TNFα + IFNγ-mediated activation of hCMEC/D3 cells increased THP-1 cell adhesion in static adhesion assays (Figure 2d and e). Pre-treatment of hCMEC/D3 cells with apabetalone (1 h) reduced THP-1 cell adhesion in the presence of cytokines, in a dose-dependent manner (18% at 5 µM and 47% at 25 µM) (Figure 2d and e).

**Figure 3:**
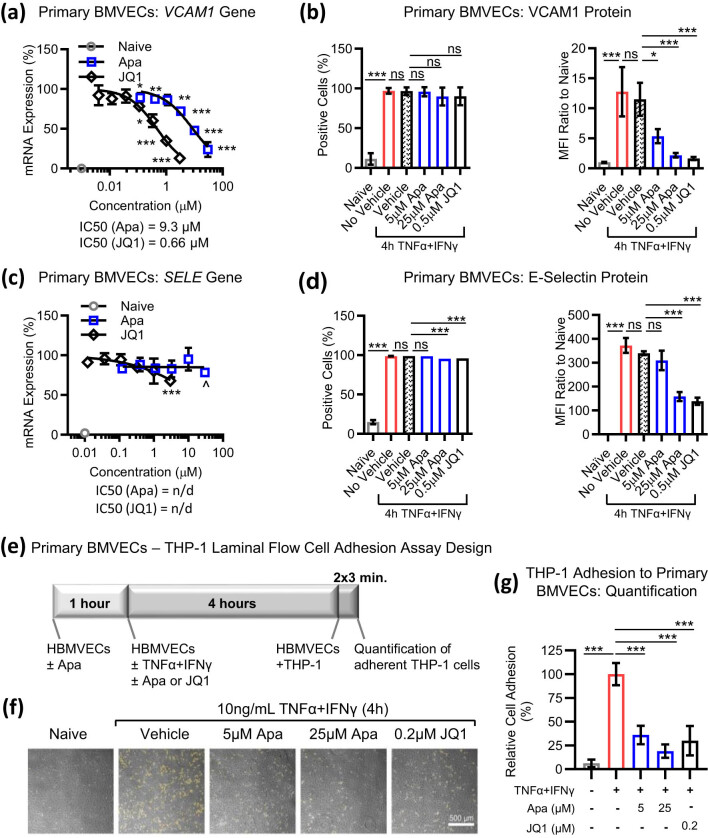
BETi reduce cytokine-induced CAM levels and monocyte adhesion in primary BMVECs. Primary BMVECs were pre-treated with apabetalone or JQ1 for 1 h prior to incubation with 10 ng/mL TNFα + IFNγ for 4 h, followed by gene expression analysis (real-time PCR) or surface expression analysis (flow cytometry) of VCAM-1 (a and b) or E-selectin (encoded by *SELE*) (c and d). Percentage of cells positive for each protein and mean fluorescence intensity (MFI) values observed in non-treated (naive) or cytokine-treated conditions are shown. The grey symbol in (a) or bars in (b) represent the non-stimulated (naive) state. 0.05% DMSO was used as the compound vehicle. Statistical analysis: one-way ANOVA with Dunnett’s multiple comparison test. (e) Experimental protocol for THP-1 cell adhesion to primary BMVEC monolayers in laminal flow conditions. (f) Phase-contrast micrographs show monocyte adhesion to primary BMVECs in the absence (naive) or presence of cytokines, DMSO, or BETi. Attached THP-1 cells are pseudocolored in yellow. Scale bar 500 μm. (g) Quantification of microscopy shows a reduction of cytokine-induced THP-1 cell adhesion to primary BMVECs in the presence of BETi (average of eight images/condition of three experimental replicates). Statistical analysis: one-way ANOVA with Dunnett’s multiple comparison test. *p ≤ 0.05; **p ≤ 0.01; ***p ≤ 0.001, ns, non-significant.

To validate the data from hCMEC/D3 cell line, we used primary human BMVECs in a laminar flow cell adhesion assay that recapitulates the effects of blood shear stress *in vitro* [32]. The capture of monocytes under shear stress conditions depends on endothelial receptors VCAM-1, ICAM-1, and E-selectin, as well as the CCL2/MPC-1 chemokine [33]. Gene and protein expression of VCAM-1 (Figure 3a and b) and E-selectin (Figure 3c and d) were upregulated by the 4 h TNFα + IFNγ treatment. Pretreatment with apabetalone or JQ1 downregulated *VCAM1* gene and protein expression by more than 80% (gene IC_50_ = 9.3 and 0.66 μM, respectively). E-selectin protein expression was not sensitive to the lower dose of apabetalone (5 μM) but showed a partial response at a higher dose (25 μM) (Figure 3d). *ICAM1* gene transcript levels, although induced by cytokines, were not sensitive to BETi, whereas *CCL2* gene expression showed a weak response to JQ1 and apabetalone (Figure S2a and b). In laminar flow cell adhesion assays (Figure 3e), primary BMVEC pretreatment with apabetalone reduced THP-1 cell adhesion by 64% at 5 µM and 81% at 25 µM (Figure 3f and g). Both 0.2 µM JQ1 and 5 µM apabetalone had a comparable effect on THP-1 adhesion to primary BMVECs (Figure 3f and g), confirming on-target treatment specificity. These data indicate that BETi affect endothelial–monocyte interactions in the presence of shear stress, possibly through downregulation of endothelial VCAM-1 and E-selectin.

### 3.4 BETi lower monocyte chemokine receptor expression, chemoattraction, and adhesion to BMVECs

**Figure 4:**
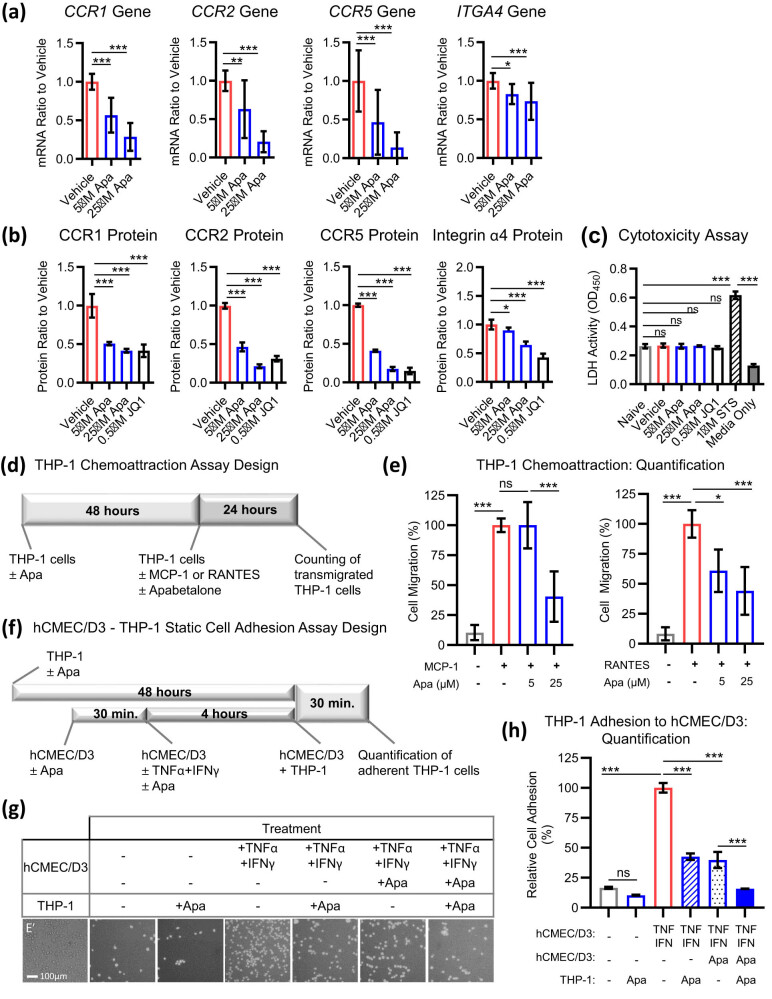
Apabetalone reduces monocyte receptor expression, *in vitro* chemoattraction, and adhesion to BMVECs. (a) Gene expression in THP-1 cells treated with vehicle or BETi for 48 h. (b) Surface protein expression in THP-1 cells treated with vehicle (DMSO) or BETi for 48 h. Median fluorescence intensity (MFI) values obtained by flow cytometry were normalized to vehicle-treated cells. (c) Vehicle (DMSO) or BETi treatment of undifferentiated THP-1 cells for 48 h did not cause cytotoxicity as measured by lactate dehydrogenase (LDH) activity in the tissue culture supernatant (expressed as optical density [OD] at 450 nm). The cytotoxic compound staurosporine (STS) served as the positive control. Media never exposed to cells (media only) displayed low LDH activity. (d) Experimental protocol for THP-1 cell chemoattraction assay. (e) The addition of MCP-1 or RANTES to the bottom chamber stimulates the migration of THP-1 cells across suspended filters. Data are presented relative to the chemokine-induced state (3 wells/condition, 3 experimental replicates). (f) Experimental protocol for THP-1 cell adhesion to hCMEC/D3 cell monolayers under static (non-flow) conditions. (g) Phase-contrast micrographs show THP-1 adhesion to hCMEC/D3 monolayers in the absence or presence of TNFα + IFNγ, DMSO or BETi. Scale bars 100 μm. (h) Quantification of cytokine-induced THP-1 static adhesion to hCMEC/D3 cells. Apabetalone pretreatment of THP-1 cells (hatched blue bar) or hCMEC/D3 cells (dotted blue bar) reduces cell–cell adhesion in the presence of cytokines by ∼60%. Apabetalone pretreatment of both hCMEC/D3 cells and THP-1 cells (solid blue bar) leads to an enhanced reduction in cell adhesion (six wells/condition, three experimental replicates). Statistical analysis: one-way ANOVA with Dunnett’s multiple comparisons test. *p ≤ 0.05; **p ≤ 0.01; ***p ≤ 0.001, ns, non-significant.

Circulating monocytes express receptors that facilitate adhesion to the activated endothelium, such as chemokine receptors CCR1, CCR2, and CCR5 [31]. In response to apabetalone (48 h), unstimulated THP-1 cells downregulated gene expression (Figure 4a) and protein surface abundance (Figure 4b) of CCR1, CCR2, and CCR5. Endothelial CAMs bind to integrin heterodimers expressed on leukocytes. The VCAM-1 ligand, integrin α4 (encoded by the *ITGA4* gene), was downregulated by apabetalone at both gene (Figure 4a) and protein (Figure 4b) levels. The viability of THP-1 cells was not affected by exposure to apabetalone for up to 48 h (Figure 4c).

To assess the functional impact of chemokine receptor downregulation on monocyte migration and adhesion, two assays were conducted. First, THP-1 cells were pretreated with apabetalone for 48 h to alter the surface expression of chemokine receptors. Then, cells were placed in the top well compartment and were allowed to migrate across the suspended filter into the bottom well compartment containing soluble chemokines for 24 h (Figure 4d). Soluble MCP-1 (CCR2 ligand) or RANTES (CCR1 and CCR5 ligand) drastically increased monocyte migration across the filter (∼10-fold) (Figure 4e). Consistent with its effects on surface receptor expression, apabetalone significantly lowered THP-1 cell chemoattraction towards both chemokines (Figure 4e). In the second assay, both THP-1 cells and hCMEC/D3 were pretreated with apabetalone and then THP-1 adhesion to cytokine-activated hCMEC/D3 cells was assessed in a static assay (Figure 4f and g). Treatment of hCMEC/D3 cells with TNFα + IFNγ significantly increased THP-1 adhesion, while apabetalone pretreatment of THP-1 cells reduced it by 60% (Figure 4h, hatched blue bar). Apabetalone pretreatment of hCMEC/D3 cells (with THP-1 cells left untreated) also resulted in a 60% decrease in THP-1 adhesion (dotted blue bar). Apabetalone pretreatment of both cell types evoked an additive response, reducing cytokine-induced cell–cell adhesion to unstimulated levels (Figure 4h, compare the solid blue bar to the open grey bar). Thus, BETi treatment of both monocytes and BMVECs reduces cognate receptor–ligand pair expression and efficiently counters monocyte adhesion to endothelial monolayers *in vitro*.

### 3.5 Apabetalone reduces endothelial and myeloid marker expression in the brain of endotoxemia mice

A pharmacokinetic study determined that a 150 mg/kg dose of apabetalone resulted in approximately 1:10 drug distribution in the mouse brain *versus* plasma at 3 h post-dose, with a concentration in brain tissue equal to 2.4 µM (Table S2). Thus, apabetalone present in plasma was predicted to have a direct impact on brain vascular endothelial cells (BMVECs) and possibly an indirect effect on the brain parenchyma. In a mouse model of endotoxemia, systemically administered lipopolysaccharide (LPS) leads to the production of inflammatory mediators in both brain vasculature and parenchyma [32,33]. Since LPS does not effectively cross the BBB [34] and, at low doses, it does not impact BBB permeability [35], its effects reach BMVECs first, before impacting brain cells in the parenchyma [36]. Thus, we used this systemic inflammation mouse model to study the effects of peripheral apabetalone exposure on vascular inflammation in the brain.

Mice were injected with a single intraperitoneal low dose of 0.4 mg/kg LPS [35]. At 24 h post-injection, gene expression of 29 inflammatory markers was examined in the brain homogenate using real-time PCR (see Section 2.9 for the full list). As compared to naive mice, LPS injection significantly increased the expression of 16 proinflammatory genes, including genes that encode cytokines *Tnf*, *Il1b*, *Csf1*, *Ccl12*, *Ccl5,* and *Cxcl10*; chemokine receptors *Ccr2* and *Cx3cr1*; CAMs *Itgam*, *Itgal*, *Sele,* and *Icam1*; LPS receptor *Cd14*; and scavenger receptors *Aif1* and *Cd68* (Table 3). Pretreatment of mice with apabetalone prior to LPS injection significantly reduced transcription of *Ccr2*, *Itgal*, *Icam1,* and *Sele* genes (Table 3, 24 h post-dose), known to mediate monocyte interactions with vascular endothelial cells [31]. The expression of *Cd68*, an inflammatory marker often associated with macrophage or microglia activation, was also decreased by apabetalone treatment (Table 3). Conversely, LPS-mediated induction of *Cxcl10* was potentiated by apabetalone treatment (Table 3).

**Table 3:** Inflammatory gene expression in brain homogenates of naive, LPS-treated, and LPS + apabetalone-treated mice (24 h post-LPS and apabetalone administration)

Gene name	LPS* ratio to naive	*p*-value†	Apabetalone‡ % change	*p*-value†
*Itgal*	352	**0.02**	−97	**0.02**
*Ccr2*	3.35	**0.01**	−77	**0.007**
*Cd68*	25.8	**<0.0001**	−58	**0.005**
*Ccl5*	695	**0.006**	−54	0.1
*Sele*	57.7	**<0.0001**	−51	**0.01**
*Icam*	35.3	**<0.0001**	−37	**0.04**
*Il1b*	15.5	**0.006**	−33	0.4
*Cxcr3*	0.54	**0.01**	−31	0.4
*Tnf*	25.4	**0.002**	−26	0.5
*Aif1*	2.81	**<0.0001**	−20	0.2
*Cx3cr1*	1.34	**0.001**	−9	0.2
*Itgam*	2.28	**<0.0001**	−8	0.6
*Csf1*	1.99	**0.003**	−7	0.6
*Cd14*	2.84	**0.04**	6	1
*Cx3cl1*	0.60	**<0.0001**	6	0.8
*Ccl12*	419	**0.004**	16	0.8
*CD69*	4.39	**0.03**	34	0.4
*Cxcl10*	22.5	**0.01**	228	**0.009**

*Mean fold-change in gene expression in the LPS-treated group normalized to naive mice (*n* = 6–8 animals).

†Statistical significance was calculated using one-way ANOVA with Dunnett’s test for multiple comparisons. Boldface represents *p* < 0.05.

‡Mean percent change in gene expression in the LPS + apabetalone-treated group normalized to LPS-treated mice (*n* = 6–8 animals).

Overall, these data indicate that, despite limited access to the brain, apabetalone can counter the effect of a systemic proinflammatory stimulus on endothelial and myeloid cell markers in the brain in agreement with *in vitro* activity.

## 4 Discussion

Chronic, low-grade inflammation is a risk factor for cognitive impairment in the general population [37] and in patients with dementia [38]. Systemic inflammatory mediators activate cerebral endothelial cells, leading to increased expression of cytokines and adhesion molecules, loss of monolayer integrity, and permeability to leukocytes [2]. This endothelial phenotype contributes to neurological inflammatory diseases, making the molecular mechanisms that control endothelial activation potential therapeutic targets [1]. Here, we demonstrate that BETi can inhibit proinflammatory and chemoattractive signaling in BMVECs and THP-1 cells, leading to decreased cell–cell interactions *in vitro*. These findings agree with brain gene expression profiling in the systemic inflammation mouse model where apabetalone attenuates markers of leukocyte and endothelial inflammatory response.

To mimic apabetalone’s blood–brain distribution *in vivo*, we assessed the impact of apabetalone on cytokine secretion in filter-grown BMVEC monolayers [39,40]. In our experiments, apical stimulation with TNFα + IFNγ evoked a substantial bilateral chemokine release (Figure 1). Immobilization of secreted chemokines on apical endothelial glycosaminoglycans enhances leukocyte adhesion to vascular walls, while binding of basolateral-secreted chemokines to the subendothelial matrix promotes directional migration of leukocytes to sites of brain inflammation [41]. Basolateral-derived cytokines produced by BMVECs are also sensed by brain-resident cells (pericytes, perivascular macrophages, astrocytes, and microglia), contributing to pro-inflammatory signaling in the brain parenchyma [35,42]. Since the apical addition of apabetalone bilaterally reduced endothelial secretion of inflammatory mediators, peripheral drug exposure *in vivo* may alleviate harmful effects of endothelial activation on the neurovascular unit on both sides of the BBB.

Activated BMVECs express multiple CAMs, including selectins, integrins, integrin ligands, and CAMs, which control the capture, rolling, arrest, and transmigration of leukocytes [2]. At low and high doses, apabetalone reduced endothelial VCAM-1 expression. VCAM-1 binds to integrin α4/integrin β1 heterodimer expressed on monocytes. In THP-1 cells studied here, surface expression of the integrin α4 subunit (encoded by the *ITGA4* gene) was reduced by apabetalone (Figure 4). Thus, downregulation of both integrin α4 and VCAM-1, the cognate ligand–receptor pair, by apabetalone is consistent with the enhanced reduction of monocyte–endothelial adhesion observed *in vitro* where both cell types were pre-treated with the drug (Figure 4h, solid blue bar). Monocyte migration towards the neuroendothelium is also influenced by multiple chemokine receptors and their ligands, including MCP-1 and CCR2, MIP-1α and CCR1, RANTES and CCR1, as well as RANTES and CCR5 [43]. Downregulation of CCR2, CCR1, and CCR5 receptor abundance by apabetalone pretreatment resulted in reduced THP-1 chemotaxis towards soluble MCP-1 and RANTES *in vitro*. Given the impact on both endothelial secretion of MCP-1 and RANTES, and surface expression of their cognate receptors by monocytes, we conclude that apabetalone could reduce monocyte recruitment to the neuroendothelium in response to secreted chemokines. Additional studies with human primary cells are needed to further understand the relative contribution of apabetalone-mediated changes to monocyte–endothelial interactions.

Peripheral LPS causes vascular inflammation that, with time, can lead to proinflammatory microglial activation in proximity to brain vasculature [44]. Consistent with *in vitro* data, apabetalone reduced markers of vascular inflammation in LPS mouse brain homogenates. We observed downregulation of *Icam1* and *Sele* gene expression, known to localize to brain endothelial cells and their support cells, namely pericytes and astrocytes [42,45]. Apabetalone also robustly repressed the expression of *Ccr2* and *Itgal* genes, which are highly expressed in peripheral leukocytes but not in brain-resident microglia [46,47,48,49,50], suggesting that BETi may impact the recruitment of peripheral immune cells to the BBB during systemic inflammation. Apabetalone also reduced the expression of the macrophage and microglial gene *Cd68* (∼60%, *p* = 0.004) (Table 3), indicating a potential impact on peripheral monocyte infiltration and/or microglial activation [32]. Interestingly, relief of leukocyte crowding in brain capillaries was recently reported as a new approach to rapidly improve short-term memory in several mouse models of Alzheimer’s disease (AD) [51,52]. Additional studies are needed to better understand how systemic effects of apabetalone can impact brain vasculature and parenchyma at a cellular level.

In the clinic, apabetalone has been tested in patients with chronic disease conditions characterized by low-grade inflammation known to impact the vasculature [17,18,53]. Plasma proteomics studies have shown that 6-month apabetalone treatment beneficially lowered plasma markers of vascular inflammation in patients with CVD. Specifically, apabetalone reduced plasma levels of fractalkine, VCAM-1, and ICAM-1 (*versus* standard-of-care) [10]. Plasma proteome analysis predicted apabetalone decreased immune cell responses, including “adhesion of leukocytes”, and transcriptional signaling by TNFα, IFNγ, IL-6, GM-CSF, and IL-1β [10]. In addition, proteomics analysis of plasma from patients with chronic kidney disease predicted that apabetalone decreased cytokine activity and leukocyte movement [54]. Altogether, these data indicate that apabetalone can alter markers of chronic inflammation in patients with vascular inflammation.

Markers of vascular inflammation are expressed in the cerebral vasculature and are upregulated in patients with vascular cognitive impairment [55] and AD [51,52,56]. Treatments targeting vascular risk factors reduce the risk of developing AD and dementia and slow cognitive decline in AD patients [57,58,59,60]. Thus, apabetalone’s potential to counter vascular inflammation in patients with chronic disease uniquely positions it as a candidate therapeutic for cognitive impairment. Indeed, in the phase 3 clinical trial, apabetalone favorably impacted cognition in a subgroup of high-risk CVD patients with T2DM: subjects with a baseline Montreal cognitive assessment test score ≤21 experienced a 1.8-unit improvement after at least 12 months of apabetalone treatment (versus placebo; *p* = 0.02) [19]. Overall, data presented here provide mechanistic insights into how apabetalone treatment may reduce neuroendothelial inflammation with potential benefits for cognitive dysfunction that accompanies brain vascular disorders.

## Supplementary Material

Supplementary Figures

Supplementary Tables
