# Supplementary material for: The BET inhibitor apabetalone decreases neuroendothelial proinflammatory activation in vitro and in a mouse model of systemic inflammation
Source: Transl Neurosci. 2023 Dec 31;14(1):20220332. doi: 10.1515/tnsci-2022-0332 (PMC10787226; doi:10.1515/tnsci-2022-0332)
Supplement: Supplementary Figures [file Wasiak_et_al_Supplementary_Figures.pdf]

Wasiak et al. Figure S1 (Supporting Table 2).

Effect of MZ-1 PROTAC on BET protein target expression and on hCMEC/D3 cell viability at 24h post treatment.

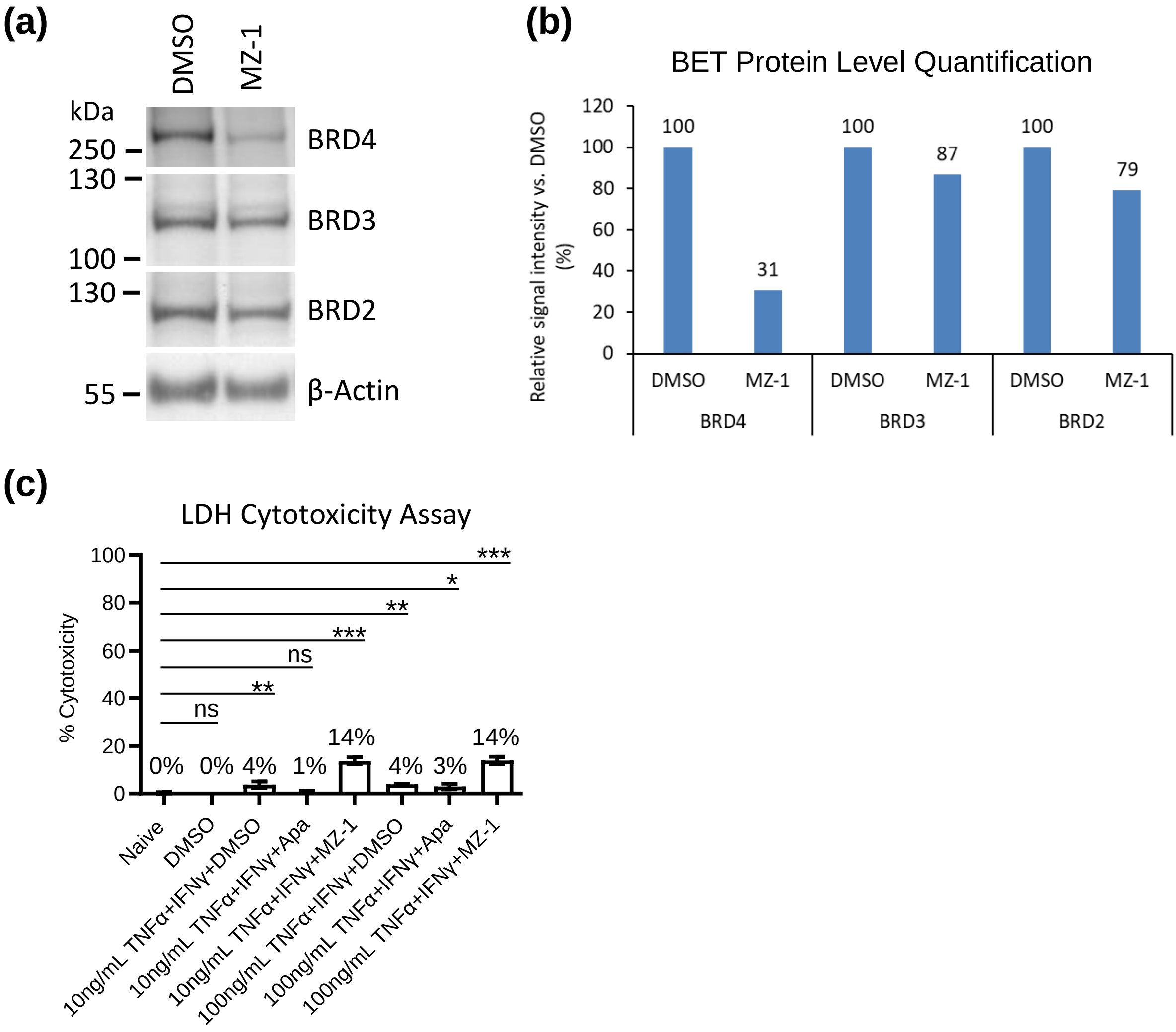

(a) 0.2µM MZ-1 (Tocris) or 0.025% DMSO was added to tissue culture media for 24h. hCMEC/D3 cell lysates were prepared in PBS + 2% SDS and sonicated. Proteins were resolved by SDS-PAGE, transferred to nitrocellulose and probed with antibodies against BRD2, BRD3 or BRD4 (Bethyl), followed by HRP-coupled secondary antibodies (Calbiochem). β-actin was detected with antibodies coupled to HRP (Sigma Aldrich). (b) Signal intensity was quantified with Quantity One software and normalized to the loading control (β-actin) and DMSO (set as 100%). (c) hCMEC/D3 cells were treated with 0.025% DMSO, MZ-1 or apabetalone in the presence or absence of TNFα + IFNγ for 24h. Tissue culture media was analyzed with LDH-Cytotoxicity Assay Kit II (Abcam). % cytotoxicity was calculated relative to lysed cells (set to 100%). Statistical analysis: one-way ANOVA with Dunnett's correction. ns, non-significant, * $p<0.05$, ** $p<0.01$, *** $p<0.001$.

Wasiak et al. Figure S2 (supporting Figure 3).
TNFα and IFNγ stimulated gene expression in primary BMVECs in the presence of BETi.

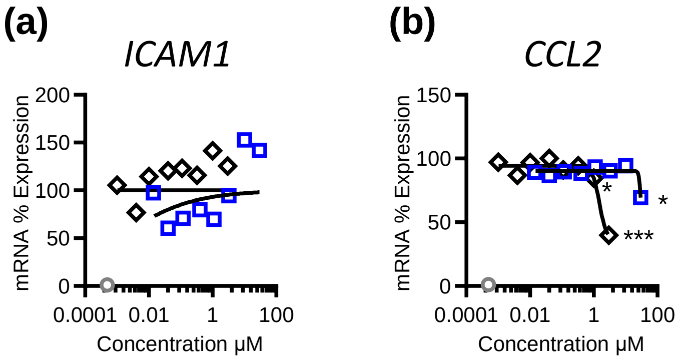

IC50 (Apa) = nd
IC50 (JQ1) = nd

IC50 (Apa) = 29 μM
IC50 (JQ1) = 1.5 μM

(a) *ICAM1* and (b) *CCL2* mRNA fold induction in response to 4h TNFα and IFNγ treatment (10 ng/mL each) was calculated relative to cytokine-naïve cells treated with vehicle (0.05% DMSO) (grey circle). Inhibition is shown relative to the induced state (set to 100%). BETi dose-response curves (green = Apa; black = JQ1) were used to calculate half inhibitory concentrations (IC50; GraphPad Prism 10). Statistical analysis: one-way ANOVA, Dunnett's multiple comparison test, where * $p<0.05$, *** $p<0.001$.
