# Supplementary material for: The BET inhibitor apabetalone decreases neuroendothelial proinflammatory activation in vitro and in a mouse model of systemic inflammation
Source: Transl Neurosci. 2023 Dec 31;14(1):20220332. doi: 10.1515/tnsci-2022-0332 (PMC10787226; doi:10.1515/tnsci-2022-0332)
Supplement: Supplementary Tables [file Wasiak_et_al_Supplementary_Tables.pdf]

Wasiak et al. Table S1 (Supporting Figure 1)

Cytokine-induced gene expression in hCMEC/D3 cells is reduced by BET protein inhibition after 24h of treatment.

Target Name		TNFα + IFNγ	TNFα + IFNγ + Apabetalone	
Protein	Gene	Fold Induction*	% Inhibition†	p-value‡
MCP-3	*CCL7*	149	96	<0.0001
Fractalkine	*CX3CL1*	964	89	<0.0001
MCP-1	*CCL2*	15	66	<0.0001
RANTES	*CCL5*	593	83	<0.0001
IL-6	*IL6*	66	81	<0.0001
IL-8	*CXCL8*	32	60	<0.0001
IP-10	*CXCL10*	14073	58	<0.0001
GM-CSF	*CSF2*	17	89	<0.0001

*mRNA fold induction in response to 24h cytokine treatment (100ng/mL) was calculated relative to cytokine-naïve cells treated with vehicle for the same amount of time (0.05% DMSO).

†Gene expression inhibition was calculated relative to the induced state in cells co-treated with cytokines and 25µM apabetalone.

‡Statistical significance was calculated with one-way ANOVA with Dunnett's correction. ns, non-significant, ns: non-significant, n=3.

Wasiak et al. Table S2 (Supporting Table 3)
Pharmacokinetic distribution of apabetalone in mouse plasma and brain tissue.

Tissue*	Mean Concentration (µM)
	Apabetalone
Brain	2.4
Plasma	32
Brain to Plasma Ratio	0.07

*Plasma and saline-perfused brains were collected from naïve mice at 3h post single oral dose of 150mg/kg apabetalone, n=3.
